# *Legionella* pneumonia in the Niagara Region, Ontario, Canada: a case series

**DOI:** 10.1186/s13256-016-1105-2

**Published:** 2016-12-01

**Authors:** Stephanie Cargnelli, Jeff Powis, Jennifer L. Y. Tsang

**Affiliations:** 1 Michael G. DeGroote School of Medicine, Niagara Regional Campus, McMaster University, MDCL 3107, 1280 Main Street West, Hamilton, ON L8S 4K1 Canada; 2 Toronto East General Hospital, 825 Cowell Avenue, Toronto, ON M4C 3E7 Canada; 3 Niagara Health, 1200 Fourth Avenue, St. Catharines, ON L2S 0A9 Canada

**Keywords:** *Legionella pneumophila*, Legionnaires’ disease, Community-acquired pneumonia

## Abstract

**Background:**

*Legionella pneumophila*, a major cause of Legionnaires’ disease, accounts for 2–15 % of all community-acquired pneumonia requiring hospitalization and up to 30 % of community-acquired pneumonia requiring intensive care unit admission. Early initiation of appropriate antimicrobial therapy is a crucial step in the prevention of morbidity and mortality. However, recognition of Legionnaires’ disease continues to be challenging because of its nonspecific clinical features. We sought to describe hospitalized community-acquired Legionnaires’ disease to increase awareness of this important and potentially lethal disease.

**Methods:**

A retrospective multicenter observational study was conducted with all patients with confirmed Legionnaires’ disease in the Niagara Region of the Province of Ontario, Canada, from June to December 2013.

**Results:**

From June to December 2013, there were 14 hospitalized cases of Legionnaires’ disease in the Niagara Region. Of these, 86 % (12 patients) had at least one comorbidity and 71 % (10 patients) were cigarette smokers*.* In our cohort, Legionnaires’ disease was diagnosed with a combination of a urinary *Legionella* antigen test and a *Legionella* real-time polymerase chain reaction assay. Delay in effective antimicrobial therapy in the treatment of *Legionella* infection led to clinical deterioration. The majority of patients had met systemic inflammatory response syndrome criteria with fever >38 °C (71 %), heart rate >90 beats per minute (71 %), and respiratory rate >20 breaths per minute (86 %). Eleven patients (79 %) required admission to the intensive care unit or step-down unit, and nine patients (64 %) required intubation. Clinical improvement after initiation of antimicrobials was protracted.

**Conclusions:**

Legionnaires’ disease should be considered during the late spring and summer months in patients with a history of tobacco use and various comorbidities. Clinically, patients presented with severe, nonspecific, multisystem disease characterized by shortness of breath, abnormal vital signs, and laboratory derangements including hyponatremia, elevated creatine kinase, and evidence of organ dysfunction. In addition, antimicrobial therapy with newer macrolides or respiratory fluoroquinolones should be initiated for severe community-acquired pneumonia requiring intensive care unit admission, prior to laboratory confirmation of diagnosis, especially when a clinical suspicion of *Legionella* infection exists.

## Background

*Legionella pneumophila*, a gram-negative bacteria and the main causative agent of legionellosis, was first recognized in 1976 [1]. Legionellosis has two distinct clinical presentations: (1) Pontiac fever, a self-limited, febrile, flulike illness; and (2) Legionnaires’ disease. Legionnaires’ disease is an atypical pneumonia that has clinical and radiographic findings similar to those of pneumococcal pneumonia [2–6]. It has an incubation period of 2–14 days. Patients might present with fever, cough, myalgia, asthenia, anorexia, and relative bradycardia [2]. Symptoms that are more suggestive of Legionnaires’ disease include gastrointestinal symptoms (diarrhea, nausea, vomiting, and abdominal pain) and neurologic symptoms (headache, obtundation, seizures, and focal neurologic findings) [2, 7–10]. Legionnaires’ disease is also characterized by nonspecific laboratory findings such as hyponatremia; hypophosphatemia; leukocytosis with relative lymphopenia; elevated creatine kinase, erythrocyte sedimentation rate, C-reactive protein, and ferritin levels; myoglobulinuria; and microscopic hematuria [2].

Legionnaires’ diseases accounts for 2–15 % of hospitalized community-acquired pneumonia (CAP) and up to 30 % of CAP requiring intensive care unit (ICU) admission [11, 12]. The incidence of Legionnaires’ disease rose by 192 % in the United States between 2000 and 2009 to 11.5 cases per 1 million population [13]. Sixty-two percent of the cases occur in the summer and autumn seasons. This is due to the increased use of air-conditioning systems and cooling towers as well as to increased rainfall [14, 15]. Approximately 20 % of cases were travel-associated [16]. Only 4 % of cases were associated with a known outbreak [2]. The risk factors for Legionnaires’ disease include cardiopulmonary disease, cigarette smoking, age >50 years, diabetes, malignancy, and immunosuppressive state including glucocorticoid use [11, 17].

Legionnaires’ disease causes significant morbidity and carries a mortality rate of 8–12 % [18]. Among patients who survive, recovery is often slow, and they are left with fatigue, neurologic and neuromuscular symptoms, and post-traumatic stress disorder [19]. Legionnaires’ disease is also very costly, with a healthcare-associated cost of more than $23,000 per case [20].

The diagnosis of Legionnaires’ disease can be made using non-culture- and culture-based techniques [2]. A urinary antigen test that detects a component of the cell wall lipopolysaccharide is the first-line diagnostic test for Legionnaires’ disease [21, 22]. However, it detects only *L. pneumophila* serogroup 1, which is the most virulent and most common cause of disease [11, 22, 23]. In Europe, >90 % of Legionnaires’ disease cases were diagnosed by urinary antigen detection [2]. It is a rapid test (results available within hours), with a sensitivity of 56–99 % [22], and it is positive within 48–72 h of symptom onset and can remain positive for several weeks or months despite treatment [21, 22]. In addition to urinary antigen detection, molecular tests such as a real-time polymerase chain reaction (PCR) assay that targets the 23S-5S ribosomal RNA (rRNA) intergenic spacer region can be used for rapid detection, and this test has the capability of differentiating *L. pneumophila* from 50 non-*L. pneumophila* species [24].

The isolation of *Legionella* bacteria by culture of the lower respiratory tract using buffered charcoal yeast extract (BCYE) medium is still the gold standard for detecting Legionnaires’ disease [22] because it allows the diagnosis of all *Legionella* species, outbreak investigation, further epidemiologic studies, and antimicrobial susceptibility testing [2]. However, culture of *Legionella* is very cumbersome and technically demanding, and the sensitivity is only around 20–80 % [2, 22].

*Legionella* species are intracellular pathogens; therefore, antibiotics should accumulate and be bioactive within the cells [2]. Azithromycin, doxycycline, or levofloxacin can be considered as first-line therapy [2]. It is recommended that levofloxacin 750 mg daily for 5–10 days or azithromycin 500 mg daily for 3–5 days be used in patients with Legionnaires’ disease, except for patients with immunosuppression, severe disease, empyema, and extrapulmonary infection, as well as those undergoing inappropriate initial therapy, for whom an extended course is recommended [25–28]. Early initiation of appropriate therapy is important in reducing mortality associated with Legionnaires’ disease [2, 29, 30].

In the summer of 2013, there were 14 hospitalized cases of Legionnaires’ disease in the Niagara Region of the Province of Ontario, Canada. The primary aim of this study was to describe these14 cases of hospitalized community-acquired Legionnaires’ disease to increase awareness of this important and potentially lethal disease.

## Methods

### Study design

We conducted a multicenter retrospective observational study of all patients with hospitalized Legionnaires’ disease in the Niagara Region of the Province of Ontario, Canada, between June and December 2013. Legionnaires’ disease was defined as a positive urinary *Legionella* antigen assay result in the presence of symptoms or *Legionella* real-time PCR of sputum or bronchoalveolar lavage (BAL) (both performed in the Public Health Ontario Laboratory). The urinary *Legionella* antigen assay was performed using a commercial Binax immunochromatographic test kits (Alere, Orlando, FL, USA) [31]. The results were reported as positive or negative. The Binax test has >70 % sensitivity and >99 % specificity for *L. pneumophila* serogroup 1 only [31]. The real-time PCR assay used in this study targets the 23S-5S rRNA intergenic spacer region. It has the ability to differentiate *L. pneumophila* from 50 other non-*L. pneumophila* species. The detailed protocol was described elsewhere [24]. All positive real-time PCR samples were cultured and identified to the genus, species, and serogroup levels using BCYE and buffered polymyxine B, anisomycin, and vancomycin charcoal media. The plates were reincubated at 35 °C for up to 14 days and examined under a microscope daily for colonial morphology such as mottled surface and iridescent red-blue-green sheen or a faceted cut-glass appearance. Slide agglutination testing was used to speciate and serogroup *L. pneumophila* isolates and atypical *Legionella*-like isolates. Known antibodies were mixed with culture isolates on a glass microscope slide. Antibodies against 50 *Legionella* species/serogroup targets were used for this method on the basis of the protocol developed by the Centers for Disease Control and Prevention in the United States [32]. Agglutination reactions were scored on a scale from 4+ (strongest) to 1+ (barely visible). In cases of cross-reaction (two or more species/serogroup targets reactive), results were compared against specific positive and negative controls. The strongest reaction was considered the final result.

### Data collection

Our data collection form encompassed demographic data, epidemiologic data, medical history, symptoms, vital signs, and laboratory test results at admission. We also recorded daily laboratory test results, antimicrobial therapy information, and clinical outcome data when available. Data collection was conducted at three sites of Niagara Health where cases occurred, including the St. Catharines sites, the Greater Niagara General Hospital site, and the Welland site. Data collection was performed using electronic medical records (Meditech, Westwood, MA, USA; IMPAX, Agfa Health Care, Greenville, SC, USA) and paper medical charts. Approval with a waiver of informed consent for the study was obtained from the Niagara Health Research Ethics Board.

## Results

### Demographics and diagnosis

From June 2013 to December 2013, there were a total of 14 hospitalized Legionnaires’ disease cases identified in the Niagara Region of Ontario, Canada*.* These contributed to approximately 2 % of all hospitalized CAP. Of note, the average number of legionellosis cases reported by Niagara Health annually from 2011 to 2015 was 6. Public Health Ontario performed a thorough evaluation of these cases and indicated that there were no epidemiological links.

Twelve (86 %) of the fourteen Legionnaires’ disease cases were identified with a positive urinary *Legionella* antigen assay. One case was tested negative with the urinary *Legionella* antigen assay due to non-*L. pneumophila* infection confirmed with BAL real-time PCR assay. One case did not have a urinary *Legionella* antigen assay sent but was tested positive for *L. pneumophila* with BAL real-time PCR assay. In total, seven (50 %) cases were tested positive with the real-time PCR assay, and three cases (21 %) were culture-positive (Table 1).

**Table 1:** Legionnaires’ disease diagnostic tests

Patient	Urinary antigen test	BAL real-time PCR	BAL culture	Sputum real-time PCR	Sputum culture
1	Positive	Negative	Negative	N/A	N/A
2	N/A	Positive	Negative	N/A	N/A
3	Positive	N/A	N/A	N/A	N/A
4	Positive	N/A	N/A	N/A	N/A
5	Positive	N/A	N/A	N/A	N/A
6	Positive	Positive	Negative	N/A	N/A
7	Positive	N/A	N/A	N/A	N/A
8	Positive	N/A	N/A	Positive	Positive
9	Positive	N/A	N/A	Positive	Positive
10	Negative	Positive^a^	Negative	N/A	N/A
11	Positive	N/A	N/A	Positive	Negative
12	Positive	N/A	N/A	N/A	N/A
13	Positive	N/A	N/A	N/A	N/A
14	Positive	Positive	Positive	N/A	N/A

*N/A* Not applicable, *BAL* bronchoalveolar lavage, *PCR* polymerase chain reaction

^a^Non-*Legionella pneumophila*

Demographics, medical comorbidities and risk factors are presented in Table 2. The majority (12 patients, 86 %) of the patients had comorbidities, with hypertension, dyslipidemia, and diabetes mellitus occurring in more than half of the cohort. In addition to the presence of comorbidities, the other major risk factors for Legionnaires’ disease in this patient population included cigarette smoking (ten patients [71 %]) and marijuana use (three patients [21 %]).

**Table 2:** Characteristics, comorbidities, risk factors, symptoms, and vital signs

Variable	Value
Age, years, median (IQR)	57 (47–66)
Male sex, *n* (%)	8 (57)
Known comorbidities/risk factors, *n* (%)	
Hypertension	9 (64)
Dyslipidemia	7 (50)
Diabetes	4 (29)
Coronary artery disease	3 (21)
Chronic lung disease	2 (14)
Chronic renal insufficiency	4 (29)
Immunosuppression	2 (14)
Cigarette smoking	10 (71)
Cannabis use	3 (21)
Symptoms, *n* (%)	
Productive cough	7 (50)
Nonproductive cough	5 (36)
Shortness of breath	12 (86)
Pleuritic chest pain	3 (21)
Nonpleuritic chest pain	2 (14)
Diarrhea	4 (29)
Nausea/vomiting	3 (21)
Abdominal pain	4 (29)
Confusion/altered level of consciousness	7 (50)
Headache	3 (21)
Fatigue	4 (21)
Rigors	7 (50)
Myalgias	4 (29)
Vital signs at triage, *n* (%)	
Heart rate >90 beats per minute	10 (71)
Respiratory rate >20 breaths per minute	12 (93)
Systolic blood pressure <90 mmHg	1 (7)
Temperature <36 °C or >38 °C	10 (71)
Laboratory values (normal range), mean ± SD	
Leukocytes (4.0–11.0), ×10^9^/L	14.9 ± 4.9
Creatinine (46–92), μmol/L	286 ± 460
Sodium (135–145), mmol/L	133 ± 4
Phosphate (0.8–1.45), mmol/L	1.48 ± 1.98
Creatine kinase (40–200), U/L	1914 ± 2728
Bilirubin (3–22), μmol/L	13 ± 11
Aspartate aminotransferase (10–40), U/L	77 ± 63
Alanine aminotransferase (5–68), U/L	54 ± 27
Alkaline phosphatase (50–135), U/L	90 ± 31
Troponin (<0.045), μg/L	0.66 ± 1.43
Lactate (0.4–2.1), mmol/L	2.3 ± 1.3
Arterial pH (7.35–7.45)	7.32 ± 0.14
Distribution of infiltrates on initial chest x-ray, *n* (%)	
Unilateral	9 (64)
Bilateral	5 (36)

### Clinical features

All 14 patients met systemic inflammatory response syndrome criteria (Table 2). Twelve patients (86 %) reported cough, with 50 % of the patients reporting sputum production. Only a minority of patients presented with gastrointestinal symptoms. Confusion or a change in mental status was present in 50 % of patients at presentation.

Laboratory results were recorded for patients upon presentation to the emergency department and are provided in Table 2. The most common laboratory abnormalities included an elevated leukocyte count (71 %), elevated creatine kinase (50 %), hyponatremia (64 %), elevated creatinine (64 %), elevated troponins (64 %), and elevated liver enzymes (86 %). At the time of admission, only five patients (36 %) had bilateral infiltrates visualized by chest x-ray.

The Pneumonia Severity Index (PSI) is a prediction rule for prognosis that objectively stratifies patients with CAP into quintiles of risk for short-term mortality on the basis of 20 demographic and clinical variables routinely available at presentation. It has been validated with data on >50,000 patients [33]. PSI scores were calculated for each patient on admission (Table 3). The majority of patients had a PSI of grade IV or above (ten patients [71 %]).

**Table 3:** Treatment, clinical complications, and outcomes

Variable	Value
Pneumonia Severity Index	
Score, mean ± SD	115 ± 45
Grade I, *n* (%)	1 (7)
Grade II, *n* (%)	0 (0)
Grade III, *n* (%)	3 (21)
Grade IV, *n* (%)	4 (29)
Grade V, *n* (%)	6 (43)
Delay in appropriate antimicrobial therapy, *n* (%)	4 (29)
Clinical complications, *n* (%)	
Vasopressor requirement	8 (57)
Mechanical ventilation	9 (64)
Renal replacement therapy	5 (36)
ICU/step-down unit admission	11 (79)
Outcomes	
Duration of mechanical ventilation, days, mean ± SD	9 ± 4
ICU/step-down unit length of stay, days, mean ± SD	13 ± 8
Hospital length of stay, days, mean ± SD	16 ± 10
Hospital mortality, *n* (%)	3 (21)

*ICU* Intensive care unit

### Treatment, clinical complications and outcomes

The treatment, clinical complications, and outcomes of the 14 patients with Legionnaires’ disease are summarized in Table 3. Delayed administration of appropriate antibiotic treatment with azithromycin or a fluoroquinolone (defined as not commencing antibiotics within 24 h after the patient presented to the emergency department) occurred in four patients. Eleven patients (79 %) required admission to an ICU or a step-down unit. The step-down unit in Niagara Health is a monitored unit with a nurse-to-patient ratio of 1:2. All patients undergo continuous cardiac monitoring and oximetry. This unit manages patients who require noninvasive ventilatory support and vasopressive support. Nine patients (64 %) required mechanical ventilation, eight patients (57 %) required vasopressive support, and five patients (36 %) required renal replacement therapy. Clinical improvement was protracted, with average ICU/step-down unit and hospital lengths of stay of 13 and 16 days, respectively. The hospital mortality rate was 21 % in this cohort.

## Discussion

Legionnaires’ disease is a life-threatening condition that requires prompt diagnosis and treatment. In this study, 64 % of the reported cases occurred from June to August 2013. This is typical in temperate climates in the northern hemisphere because the causative organism multiplies effectively in warm aquatic environments [2]. The median age of our patient cohort was 58 years, which was less than what was published by Public Health Ontario in 2011. Indeed, most of our patients were <65 years of age (71 %), despite the fact that the incidence of Legionnaires’ disease is reported to increase with age [34]. This suggests that age may not be a prominent characteristic for community-acquired Legionnaires’ disease that is not associated with a point source outbreak. Tobacco use is a well-documented risk factor for Legionnaires’ disease [35], and our results showed that cigarette smoking was a common feature in our cohort of patients with Legionnaires’ disease. A less-established risk factor for Legionnaires’ disease is cannabis use. Results published by Nguyen *et al.* provided support for an increased risk of Legionnaires’ disease in cannabis smokers and suggested that this risk may be cumulative with the risk incurred through tobacco use [36]. Interestingly, in our cohort of patients with Legionnaires’ disease, we found that three patients were both cannabis and cigarette smokers.

Clinical recognition of Legionnaires’ disease is made difficult by the fact that patient presentation is often inconsistent with the clinical presentation documented in the literature [2, 6]. Our data suggest that the diagnosis of Legionnaires’ disease on the basis of clinical presentation is unreliable.

Our results demonstrate that Legionnaires’ disease results in severe pneumonia, as reflected by the high PSI score and impacts on multiple organ systems rather than being confined solely to the pulmonary system. The multisystem nature of Legionnaires’ disease likely contributes in part to the acute presentation of patients with Legionnaire’s disease and the need for more intensive management. With almost three-fourths of the patients requiring admission to an ICU, almost two-thirds requiring mechanical ventilation for an average of 9 days, and over half requiring vasopressors, these results certainly corroborate the data suggesting that the clinical manifestations of Legionnaires’ disease are often more severe than those of pneumonias caused by other infectious agents [37].

Chest x-rays are one of the first diagnostic tests performed on patients upon arrival in the emergency department. As with much of the presentation thus far, the chest x-ray of patients with Legionnaires’ disease does not possess any distinguishing features that would guide diagnosis. The initial chest x-ray of just over one-third of the patients demonstrated bilateral airspace disease; the remaining two-thirds had unilateral findings. However, six of the patients with unilateral findings on their initial x-ray progressed to bilateral findings on follow-up images. This progression of radiographic findings throughout hospital admission prior to noting improvements has been documented in the literature [6]. There were not any identifiable patterns in terms of proclivity for specific lobes or features of lung involvement.

The most common method of Legionnaires’ disease diagnosis was urinary *Legionella* antigen testing (92.9 %). In our case series, of the 13 patients tested for urinary *Legionella* antigen, 12 had a positive result. One of the patients was infected by non-*L. pneumophila Legionella* (diagnosed with BAL real-time PCR). As such, other forms of diagnostic tests, including real-time PCR and culture of sputum or BAL samples, are indicated if there is a suspicion of Legionnaires’ disease and the result of urinary *Legionella* antigen testing is negative [38]. It has been suggested that a combination of urinary *Legionella* antigen testing and real-time PCR is the best initial approach to ensure detection of all *Legionella* infections and obtain results within a time frame capable of influencing management [39]. This is corroborated in our case series by the fact that one of the diagnoses would have been missed had a real-time PCR of a BAL sample not been done concurrently with urinary *Legionella* antigen testing.

Delay in initiating appropriate antibiotic therapy is known to be a poor prognostic factor in patients infected by *Legionella* [39]. In our case series, four patients had delayed initiation of antibiotic therapy with a fluoroquinolone or azithromycin. Although none of these patients died as a result of this delay in diagnosis and treatment, two of these patients had hospital stays that exceeded 25 days, and both of them required mechanical ventilation and vasopressor support. One patient also required dialysis throughout the admission, and three patients required admission to the ICU. As such, it is evident that, while a delay in treatment did not result in a higher mortality rate, it has the potential to worsen disease severity and result in a need for more intensive management. Of note, the three patients who died were older (aged 66, 74, and 81 years, respectively). One patient was immunosuppressed on methotrexate and prednisone for rheumatoid arthritis; two patients had a significant cardiac history and diabetes; and two patients were cigarette smokers.

A study conducted in Switzerland demonstrated that patients infected with atypical pathogens, including *Legionella*, had clinical instability refractory to treatment with a β-lactam and required the addition of a macrolide to the treatment regimen [40]. The same study demonstrated that patients with more severe pneumonias benefited from a combination therapy as opposed to β-lactam monotherapy. As such, it is suggested that the initial treatment approach for patients presenting with severe CAP (those with a PSI category IV pneumonia) include a macrolide or fluoroquinolone with effectiveness in treating *Legionella* rather than waiting for laboratory confirmation of infection [40]. Our study suggests that patients presenting with severe multisystem disease during the summer or early autumn requiring admission to the ICU need atypical coverage with a macrolide or fluoroquinolone prior to laboratory confirmation. Atypical coverage should also be added to a treatment regimen when there is no improvement noted with β-lactam treatment.

Our study was limited by the fact that our data collection was dependent on the reporting and diagnostic tests ordered by other healthcare professionals. As a result, full patient histories and laboratory values could not be obtained for each patient. A prospective study would overcome this limitation. Last, we do not have a control group with non-*Legionella* CAP to assess risk factors for Legionnaires’ disease.

## Conclusions

Legionnaires’ disease should be considered during the late spring and summer months in patients with a history of tobacco use and various comorbidities presenting with severe, multisystem disease characterized by shortness of breath; abnormal vital signs; and laboratory derangements, including hyponatremia, elevated creatine kinase, and evidence of organ dysfunction. Legionnaires’ disease should be diagnosed with a combination of urinary *Legionella* antigen testing and *Legionella* real-time PCR if available. Antibiotic therapy with a macrolide or a fluoroquinolone should be initiated for severe CAP prior to laboratory confirmation of diagnosis, especially when a clinical suspicion exists.

## Abbreviations

BAL: Bronchoalveolar lavage
BCYE: Buffered charcoal yeast extract
CAP: Community-acquired pneumonia
ICU: Intensive care unit
PCR: Polymerase chain reaction
PSI: Pneumonia Severity Index
rRNA: Ribosomal RNA
